# Does Chronological Age Adequately Stratify Perioperative Risk? A Prospective Multicenter Cohort Study Using Frailty and Handgrip Strength

**DOI:** 10.3390/jcm15114187

**Published:** 2026-05-28

**Authors:** Sergii Girnyi, Virginia Boccardi, Elena Montanari, Eugenia Semeraro, Alessandra Marano, Mauro Santarelli, Silvia Malerba, Francesco Paolo Prete, Mario Testini, Jaroslaw Skokowski, Tomasz Cwalinski, Mathias Schlögl, Mahmoud Al-Balas, Luigi Marano

**Affiliations:** 1 Department of General Surgery and Surgical Oncology, “Saint Wojciech” Hospital, “Nicolaus Copernicus” Health Center, 80462 Gdańsk, Poland; sgirnyi@copernicus.gda.pl (S.G.); tcwalinski@copernicus.gda.pl (T.C.); 2 Section of Gerontology and Geriatrics, Department of Medicine and Surgery, University of Perugia, 06132 Perugia, Italy; virginia.boccardi@unipg.it; 3 General and Specialistic Surgery Department, Emergency General Surgery Unit, A.O.U. Città della Salute e della Scienza di Torino, 10121 Turin, Italy; emontanari@cittadellasalute.to.it (E.M.); esemeraro@cittadellasalute.to.it (E.S.); amarano@cittadellasalute.to.it (A.M.); msantarelli@cittadellasalute.to.it (M.S.); 4 Department of Precision and Regenerative Medicine and Ionian Area, University of Bari “Aldo Moro”, 70100 Bari, Italy; s.malerba10@studenti.uniba.it (S.M.); francesco.prete@uniba.it (F.P.P.); mario.testini@uniba.it (M.T.); 5 Department of Medicine, Academy of Applied Medical and Social Sciences-AMiSNS (Akademia Medycznych I Spolecznych Nauk Stosowanych), 82300 Elbląg, Poland; j.skokowski@amisns.edu.pl; 6 Department for Geriatric Medicine, Clinic Barmelweid AG, 5012 Barmelweid, Switzerland; mathias.schloegl@barmelweid.ch; 7 Department of General Surgery, Anesthesia and Urology, Faculty of Medicine, The Hashemite University, Zarqa 13133, Jordan; mahmoud_albalas@hu.edu.jo; 8 Department of Surgery, Dnipro State Medical University, 49000 Dnipro, Ukraine

**Keywords:** frailty, handgrip strength, length of stay, perioperative risk stratification, abdominal surgery

## Abstract

**Background**: Chronological age remains deeply embedded in perioperative risk assessment because it is readily available and intuitively associated with adverse outcomes. In clinical practice, however, patients of similar age frequently experience markedly different postoperative trajectories, suggesting that physiological reserve may more accurately reflect vulnerability to surgical stress than years lived alone. We therefore investigated whether age-based stratification inadequately captures perioperative vulnerability when compared with functional phenotyping based on frailty status and baseline handgrip strength (HGS). **Methods**: We conducted a prospective multicenter observational cohort study including 223 adults undergoing elective abdominal surgery between January 2023 and June 2025. Chronological age was evaluated both continuously and using a conventional threshold (<70 vs. ≥70 years). Physiological reserve was characterized using a phenotype-based frailty model (fit, pre-frail, frail) and baseline HGS measured at hospital admission. Prolonged hospitalization, defined a priori as length of stay (LOS) > 10 days, was used as a pragmatic clinical benchmark. Cross-classification analyses, logistic regression, and receiver operating characteristic (ROC) curve analyses were performed to compare the discriminatory performance of chronological age, frailty phenotype, and HGS. **Results**: Substantial discordance was observed between chronological age and frailty phenotype. Among patients younger than 70 years, 7.7% met criteria for frailty, whereas 58.0% of patients aged ≥70 years were classified as fit or pre-frail. Prolonged hospitalization occurred in 48 patients (21.5%) and varied markedly according to frailty status within each age group. Frail patients younger than 70 years demonstrated higher rates of prolonged LOS than fit older patients (40.0% vs. 10.5%). Chronological age demonstrated limited discrimination for prolonged hospitalization (AUC 0.579), while the ≥70-year threshold showed poor discriminatory performance (AUC 0.541). Frailty phenotype demonstrated improved discrimination (AUC 0.679), whereas the combined multivariable model integrating age, frailty, HGS, sex, and oncologic indication achieved good discriminatory performance (AUC 0.810). In multivariable analyses, frailty remained independently associated with prolonged LOS, whereas chronological age did not. **Conclusions**: Chronological age alone demonstrated limited discriminatory performance for perioperative risk stratification. Functional phenotyping based primarily on frailty status, complemented by objective functional measures such as HGS, may better capture physiological reserve and support more individualized, function-centered perioperative assessment in abdominal surgery.

## 1. Introduction

Chronological age remains deeply embedded in perioperative decision-making because it is immediately available and intuitively associated with surgical risk. Yet, in clinical practice, patients of identical chronological age often experience markedly different postoperative trajectories, suggesting that vulnerability to surgical stress may depend less on “years lived” than on the physiological reserve with which a patient enters surgery. This discrepancy has become increasingly evident as surgical populations age and case complexity grows, raising concern that age thresholds may simultaneously overestimate vulnerability in some older adults while under-recognizing risk in younger patients with impaired reserve [1,2].

Frailty provides a clinically relevant framework to characterize this heterogeneity. Rather than reflecting comorbidity burden alone, frailty denotes a state of diminished resilience arising from multisystem dysregulation and reduced capacity to respond to stressors [3,4,5]. Across surgical specialties, frailty has consistently been associated with postoperative complications, prolonged length of stay (LOS), functional decline, and mortality [6,7,8,9,10,11]. In oncologic surgery, frailty has also been linked to recurrence and survival, suggesting that physiological vulnerability may extend beyond the immediate perioperative period [12]. Importantly, frailty is not restricted to advanced age. Studies in emergency abdominal surgery and trauma have identified frailty in younger or “non-elderly” adults, challenging the assumption that chronological age reliably partitions patients into low- and high-risk groups [13,14]. These observations have supported increasing integration of frailty assessment into perioperative pathways and shared decision-making processes rather than reliance on age cut-offs alone [15].

Two principal operational frameworks are commonly used in clinical practice. The phenotype model defines frailty through observable physical characteristics—weakness, slowness, exhaustion, unintentional weight loss, and low physical activity—that collectively reflect impaired physiological reserve [16]. In contrast, the deficit accumulation model conceptualizes frailty as the proportion of age-related deficits accumulated across multiple domains [17,18]. Although grounded in different theoretical premises, both approaches appear to capture overlapping, though not identical, dimensions of vulnerability and have demonstrated clinical relevance in surgical populations [19]. Recent perioperative guideline initiatives have further emphasized that vulnerability is distributed along a continuum rather than concentrated within a single “older” category [20].

Within these multidimensional constructs, muscle function represents a particularly informative domain. Handgrip strength (HGS) is attractive because it is inexpensive, rapid, reproducible, and reflective of integrated neuromuscular performance rather than a single disease process [21,22,23,24]. In both medical and surgical settings, lower grip strength has been associated with prolonged hospitalization and adverse outcomes [25,26,27]. In major abdominal and oncologic surgery, prospective studies and systematic reviews have similarly suggested that reduced preoperative HGS is associated with prolonged LOS and increased postoperative burden [28,29]. More recent evidence continues to support the role of preoperative grip strength as a pragmatic marker of physiological reserve rather than a purely geriatric descriptor [30].

However, most perioperative studies have primarily evaluated frailty and HGS as outcome predictors, whereas their classification performance relative to chronological age has received less direct attention [31]. This distinction is clinically important. Demonstrating that frailty or low HGS correlates with worse outcomes differs conceptually from determining whether chronological age itself provides limited discriminatory precision, potentially leading to clinically relevant risk re-ranking within age strata [32]. Age-based thresholds may therefore trigger management strategies, monitoring intensity, or resource allocation that are disproportionate to actual physiological reserve, while simultaneously failing to identify younger patients with substantial vulnerability. In parallel, contemporary perioperative quality initiatives increasingly emphasize structured assessment of geriatric syndromes and functional reserve, reflecting a broader transition toward function-centered surgical care [31]. Implementation studies related to geriatric surgery programs and age-friendly surgical pathways further illustrate how structured assessment frameworks can be operationalized at the system level, reinforcing the concept that perioperative risk in older adults cannot be adequately reduced to chronological age alone [33,34].

Against this background, prospective multicenter data examining chronological age and functional phenotyping through the lens of discordance, overlap, and risk re-ranking in abdominal surgery remain limited. Previous studies have suggested that grip strength may provide information beyond age and frailty, although most available analyses have been retrospective or primarily focused on outcome prediction [31]. In the present prospective multicenter cohort, we approached the problem primarily as a methodological question. We quantified discordance between chronological age, including a conventional 70-year threshold, and frailty phenotype; characterized within-age dispersion and cross-age overlap in baseline HGS; and used prolonged hospitalization as a pragmatic clinical benchmark to illustrate risk re-ranking across age–frailty strata. By focusing on classification behavior rather than causal inference, we aimed to clarify whether chronological age alone provides limited discriminatory precision and whether functional phenotyping offers a more clinically coherent framework for perioperative stratification.

## 2. Materials and Methods

### 2.1. Study Design and Ethical Considerations

We conducted a prospective multicenter observational study designed to examine how different perioperative stratification frameworks—specifically chronological age and functional phenotyping—classify surgical risk. Adult patients undergoing elective abdominal surgery were consecutively enrolled between February 2023 and June 2025 at participating academic centers.

All participants provided written informed consent. The protocol complied with the Declaration of Helsinki and Good Clinical Practice standards and received approval from the coordinating institutional ethics committee (AOU Città della Salute e della Scienza di Torino; protocol 0025277, approved on 27 February 2023). Reporting followed the Strengthening the Reporting of Observational Studies in Epidemiology (STROBE) recommendations for observational research [35].

### 2.2. Study Population

Eligible individuals were adults (≥18 years) scheduled for elective abdominal procedures requiring postoperative hospitalization. To reduce heterogeneity unrelated to physiological reserve, we excluded diagnostic or exploratory procedures, palliative interventions, day-case surgery, and patients unable to provide informed consent.

Because functional reserve represented a central methodological focus, availability of baseline HGS assessment at hospital admission was required for analyses involving muscle strength. Patients without baseline HGS measurements were excluded only from strength-based analyses.

### 2.3. Frailty Phenotyping

Physiological vulnerability was characterized using a phenotype-based frailty model emphasizing physical reserve rather than comorbidity burden [16]. Five domains were assessed preoperatively: unintentional weight loss, self-reported exhaustion, reduced physical activity, slowness, and muscle weakness.

Unintentional weight loss was defined as self-reported involuntary loss of >4.5 kg or >5% of body weight during the previous year. Exhaustion was assessed through patient-reported persistent fatigue, reduced endurance, or impaired ability to perform usual daily activities. Reduced physical activity was defined clinically according to self-reported limitation of habitual mobility, reduced participation in regular exercise, or decreased engagement in routine physical activities compared with baseline status. Slowness was assessed clinically based on reduced habitual walking performance, mobility limitation, or visibly impaired gait speed during routine ambulation. Muscle weakness was evaluated using baseline HGS assessment performed at hospital admission.

Each frailty component contributed one point, generating a cumulative frailty score ranging from 0 to 5. Participants were classified as fit (0 points), pre-frail (1–2 points), or frail (3–5 points), consistent with the original phenotype model. Frailty assessments were performed preoperatively by trained investigators according to standardized procedures across participating centers.

### 2.4. Handgrip Strength Assessment

Handgrip strength was measured using a digital dynamometer (EH101, Deyard, Shenzhen, China) according to standardized procedures consistent with American Society of Hand Therapists (ASHT) recommendations [36]. Assessments were performed with participants seated, shoulder adducted, elbow flexed at 90°, and wrist in slight extension.

Following three familiarization contractions, participants performed three maximal voluntary isometric contractions with the dominant hand, and the highest value was retained for analysis. HGS was assessed at admission, discharge, and 90 days postoperatively in a subset of patients with complete follow-up. However, the present analyses focused specifically on baseline HGS as a perioperative stratification variable.

### 2.5. Benchmark Clinical Variables

Postoperative LOS was not treated as the primary outcome, but rather as a pragmatic clinical benchmark against which stratification performance could be evaluated.

LOS was defined as the number of days from surgery to discharge. Prolonged hospitalization was specified a priori as LOS > 10 days, corresponding to the 75th percentile of the cohort distribution. Although LOS may be influenced by organizational and discharge-related factors, it was selected as a pragmatic cumulative marker of postoperative burden commonly used in perioperative research. Postoperative complications were prospectively recorded and graded according to the Clavien–Dindo classification [37], with major complications defined as grade ≥ III.

The cohort included heterogeneous abdominal procedures performed across multiple participating centers, including both oncologic and non-oncologic operations with variable operative complexity and perioperative recovery trajectories. Although center-specific discharge pathways, rehabilitation availability, operative approach, and perioperative management strategies were not formally modeled as covariates, these factors were considered potential contributors to residual variability in postoperative LOS and should therefore be considered when interpreting the findings.

### 2.6. Statistical Analysis

All analyses were performed using SPSS Statistics version 26 (IBM Corp., Chicago, IL, USA). The analytical strategy aimed to evaluate the discriminatory performance of chronological age, frailty phenotype, and baseline HGS for perioperative risk stratification.

Sample size was estimated a priori using G*Power software (version 3.1.9.7), assuming a small-to-moderate effect size (f^2^ = 0.10), a two-sided α level of 0.05, and 80% statistical power. A minimum sample size of 179 patients was required. The final cohort of 223 patients exceeded this threshold and was considered adequate for the planned multivariable analyses, although exploratory subgroup comparisons across combined age–frailty strata should be interpreted cautiously.

Chronological age was analyzed both as a continuous variable and using a conventional threshold (<70 vs. ≥70 years). Frailty phenotype was categorized as fit, pre-frail, or frail, whereas baseline HGS was analyzed primarily as a continuous variable. Continuous variables were summarized as mean ± standard deviation or median with interquartile range (IQR), as appropriate. Categorical variables were reported as counts and percentages.

Prolonged hospitalization was defined a priori as LOS > 10 days, corresponding to the 75th percentile of the cohort distribution, and was used as a pragmatic clinical benchmark. Associations with prolonged LOS were initially explored using univariate logistic regression analyses. Variables considered clinically relevant were subsequently entered into multivariable logistic regression models. Odds ratios (ORs) with 95% confidence intervals (CIs) were reported.

For regression analyses, frailty phenotype was entered as an ordinal categorical variable reflecting increasing physiological vulnerability (fit = 1, pre-frail = 2, frail = 3). Baseline HGS and chronological age were modeled as continuous variables, whereas sex and oncologic indication were entered as binary covariates. Because baseline HGS contributed to the operational definition of frailty phenotype, multicollinearity diagnostics were assessed using variance inflation factor (VIF) analysis. All VIF values remained below 2, suggesting absence of problematic multicollinearity among included covariates.

Receiver operating characteristic (ROC) curve analyses were performed for chronological age, age threshold (≥70 years), frailty phenotype, baseline HGS, and the combined multivariable model integrating age, frailty phenotype, HGS, sex, and oncologic indication. Area under the curve (AUC), sensitivity, specificity, positive predictive value (PPV), negative predictive value (NPV), and overall classification accuracy were calculated where appropriate. Model calibration was assessed using the Hosmer–Lemeshow goodness-of-fit test.

Baseline HGS distributions across age strata were additionally explored using descriptive and graphical analyses. Independent-samples *t* tests or Mann–Whitney U tests were used for continuous variable comparisons, as appropriate. All statistical tests were two-sided, and *p* < 0.05 was considered statistically significant.

## 3. Results

### 3.1. Cohort Characteristics and Stratification Framework

A total of 223 patients were enrolled. Chronological age spanned a broad range, while frailty phenotyping demonstrated substantial heterogeneity in physiological reserve across the cohort. Using a conventional age threshold, 130 patients were classified as <70 years and 93 as ≥70 years. In contrast, frailty phenotyping identified 37.2% of patients as fit, 40.8% as pre-frail, and 22.0% as frail, with these categories distributed across the entire age spectrum. This divergence between chronological and functional stratification frameworks informed the subsequent analyses of classification concordance. The cohort included both oncologic and non-oncologic abdominal procedures characterized by heterogeneous operative complexity and postoperative recovery trajectories (Table 1).

### 3.2. Age–Frailty Discordance and Classification Overlap

Cross-classification of chronological age and frailty status demonstrated marked discordance between age-based and functional vulnerability stratification. Among patients younger than 70 years (n = 130), nearly half were classified as fit (49.2%), while an additional 43.1% were categorized as pre-frail, leaving only 7.7% meeting criteria for frailty. Accordingly, 92.3% of patients below the conventional age threshold were not frail. In patients aged ≥70 years (n = 93), frailty status showed a more heterogeneous distribution. Although frail individuals constituted the largest subgroup (41.9%), substantial proportions of older patients were classified as pre-frail (37.6%) or fit (20.4%). The distribution of frailty categories across age groups is summarized in Table 2.

Accordingly, 58.0% of older patients were not classified as frail, suggesting that age-based thresholds may overestimate vulnerability in a substantial proportion of surgical candidates. Conversely, chronological age alone also failed to adequately identify physiological vulnerability. Among all patients classified as frail, 20.4% were younger than 70 years, indicating that reliance on chronological age alone may overlook a clinically meaningful subset of vulnerable individuals.

### 3.3. Risk Re-Ranking Across Age and Frailty Strata Using Prolonged Hospitalization

Prolonged hospitalization (LOS > 10 days) occurred in 48 patients and was used as a pragmatic clinical benchmark for classification analyses. Among patients < 70 years, prolonged hospitalization occurred in 19.2% overall. Stratification according to frailty phenotype revealed substantial within-group heterogeneity, with prolonged LOS observed in 10.9% of fit patients, 25.0% of pre-frail patients, and 40.0% of frail patients.

Among patients aged ≥70 years, the overall rate of prolonged LOS was 24.7%. Frailty phenotype again differentiated postoperative burden, with prolonged LOS occurring in 10.5% of fit patients, 11.4% of pre-frail patients, and 43.6% of frail patients. Notably, older fit and pre-frail individuals demonstrated hospitalization rates comparable to, or lower than, those observed in younger fit patients.

Direct cross-age comparisons further illustrated clinically relevant risk re-stratification. Frail patients younger than 70 years experienced a substantially higher burden of prolonged hospitalization (4/10 patients; 40.0%, 95% CI 12.2–73.8) than fit patients aged ≥70 years (2/19 patients; 10.5%, 95% CI 1.3–33.1). However, confidence intervals were relatively wide in some strata, particularly among younger frail individuals, reflecting limited subgroup sample size and indicating that these findings should be interpreted cautiously (Table 3).

Chi-squared analysis demonstrated significant differences in prolonged hospitalization rates across the combined age–frailty strata (χ^2^ = 21.12, *p* < 0.001), supporting the presence of clinically relevant heterogeneity within chronological age groups according to frailty status.

Postoperative complications demonstrated patterns broadly consistent with the LOS-based analyses, with frail individuals showing the highest burden of both overall and major postoperative complications (Supplementary Table S1). Overall postoperative complications occurred in 80 patients (35.9%), while major complications (Clavien–Dindo grade ≥ III) occurred in 15 patients (6.7%). Frail patients tended to demonstrate a greater burden of postoperative complications across age strata, whereas chronological age alone showed less consistent separation of complication risk. However, because the present study was not specifically powered for detailed complication-stratified subgroup analyses, these findings should be considered exploratory and interpreted cautiously.

Conversely, older pre-frail patients demonstrated one of the lowest observed rates of prolonged hospitalization (11.4%) despite exceeding the conventional age threshold. When chronological age alone was considered, prolonged LOS occurred in 19.2% of patients < 70 years and 24.7% of those aged ≥70 years, suggesting relatively limited discriminatory separation between age strata.

### 3.4. Discriminatory Performance of Chronological Age, Frailty, and Handgrip Strength

ROC curve analyses were performed to compare the discriminatory performance of chronological age, frailty phenotype, and baseline HGS for prolonged hospitalization (LOS > 10 days). Chronological age analyzed as a continuous variable demonstrated limited discriminatory capacity (AUC 0.579), while the conventional age threshold of ≥70 years showed poor classification performance (AUC 0.541). Frailty phenotype demonstrated improved discrimination (AUC 0.679), whereas baseline HGS alone showed only modest discriminatory performance (AUC 0.544).

**Figure 1:**
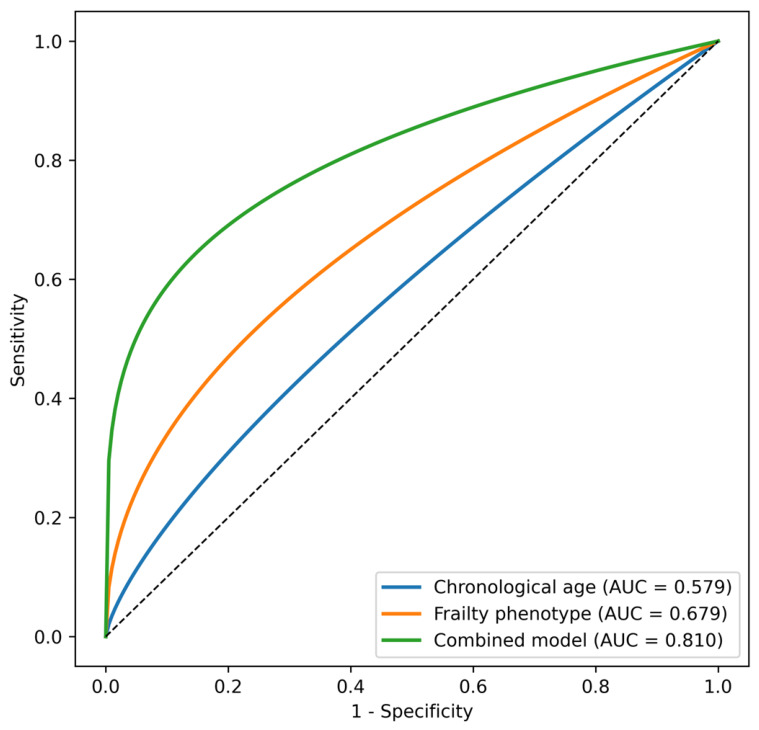
Receiver operating characteristic (ROC) curves comparing discriminatory performance of chronological age, frailty phenotype, and the combined multivariable model for prolonged hospitalization. The dashed diagonal line represents the reference line corresponding to no discriminatory ability (AUC = 0.5).

In contrast, a combined multivariable model integrating chronological age, frailty phenotype, baseline HGS, sex, and oncologic indication demonstrated substantially improved classification performance, achieving an AUC of 0.810 (Figure 1).

ROC curves showing the predictive performance of chronological age, frailty phenotype, and the combined multivariable model for prolonged postoperative hospitalization (LOS > 10 days). Chronological age showed limited discrimination (AUC 0.579), frailty phenotype performed better (AUC 0.679), and the combined model demonstrated the highest accuracy (AUC 0.810).

Classification metrics further emphasized the limited performance of age-based stratification. The ≥70-year threshold demonstrated a sensitivity of 47.9%, specificity of 59.4%, positive predictive value (PPV) of 24.7%, negative predictive value (NPV) of 80.8%, and an overall classification accuracy of 57.0% for prolonged hospitalization. In contrast, frailty-based stratification demonstrated higher specificity (86.3%), NPV (86.7%), and overall classification accuracy (78.5%) (Table 4).

In univariate logistic regression analyses, chronological age showed only a borderline association with prolonged LOS (OR 1.02 per year increase, *p* = 0.062), whereas the ≥70-year threshold was not significantly associated with prolonged hospitalization (OR 1.40, *p* = 0.305). Frailty phenotype demonstrated a strong association with prolonged LOS (OR 3.01, *p* < 0.001).

Baseline HGS demonstrated an inverse relationship with prolonged hospitalization, with lower grip strength values tending to associate with greater postoperative burden, although this association did not independently reach statistical significance in univariate analyses. Nevertheless, inclusion of HGS within the combined multivariable model contributed to the improved discriminatory performance observed for the integrated functional stratification framework.

Multivariable logistic regression analysis demonstrated that frailty phenotype remained independently associated with prolonged hospitalization after adjustment for chronological age, baseline HGS, sex, and oncologic indication (adjusted OR 3.01, 95% CI 1.65–5.46; *p* < 0.001), whereas chronological age was no longer significantly associated with prolonged LOS (adjusted OR 0.98, 95% CI 0.95–1.01; *p* = 0.232). Baseline HGS demonstrated a borderline inverse association with prolonged hospitalization (adjusted OR 0.96 per kg increase, 95% CI 0.92–1.00; *p* = 0.063). Male sex and oncologic indication were also independently associated with prolonged LOS.

The combined model demonstrated acceptable calibration according to the Hosmer–Lemeshow goodness-of-fit test (*p* = 0.72) (Table 5).

### 3.5. Within-Age Dispersion of Baseline Muscle Strength

Baseline HGS demonstrated wide dispersion within both age groups, highlighting substantial inter-individual variability in physiological function that was not fully captured by chronological age alone. Among patients younger than 70 years (n = 130), mean baseline HGS was 28.3 ± 11.1 kg, with a median of 27.2 kg and an interquartile range (IQR) of 20.6–36.3 kg. Values ranged from 6.4 kg to 60.1 kg, with the 10th and 90th percentiles at 14.7 kg and 42.5 kg, respectively.

**Figure 2:**
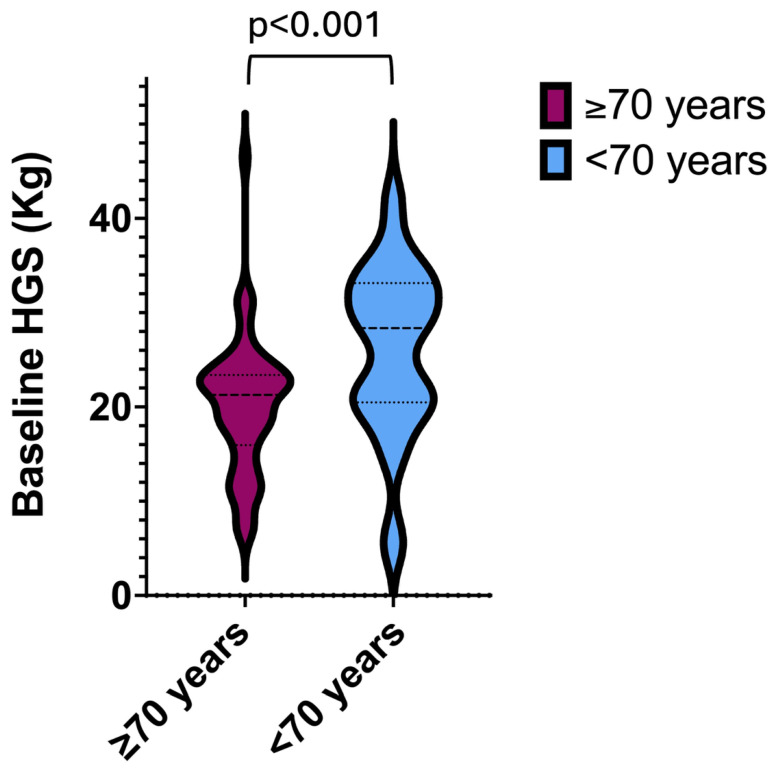
Overlap of baseline handgrip strength distributions across chronological age groups. Violin plots illustrate the distribution of baseline handgrip strength in patients younger than 70 years and those aged 70 years or older. The width of each violin reflects the density of observations. The central dashed line represents the median, while the upper and lower dotted lines indicate the interquartile range (25th and 75th percentiles). Baseline HGS differed significantly between age groups (*p* < 0.001).

In patients aged ≥70 years (n = 93), baseline HGS was lower on average (20.5 ± 9.7 kg), with a median of 19.3 kg and an IQR of 14.5–29.4 kg (Figure 2). Despite this downward shift, substantial variability persisted, with values ranging from 1.0 kg to 42.5 kg. Notably, the 75th percentile among older patients (29.4 kg) exceeded the median observed in younger patients, underscoring the marked overlap in physiological reserve across chronological age strata.

In an exploratory supplementary analysis using sex-specific HGS reference thresholds (<35 kg for males and <20 kg for females), patients with reduced baseline HGS demonstrated a numerically higher rate of prolonged hospitalization compared with individuals with preserved HGS (25.2% vs. 15.5%), although the difference did not reach statistical significance (χ^2^ = 2.37, *p* = 0.123).

## 4. Discussion

In this prospective multicenter cohort, we examined perioperative risk stratification through the combined lens of chronological age, frailty phenotype, and baseline HGS. The principal finding of the present study is that chronological age alone demonstrated limited discriminatory performance for prolonged postoperative hospitalization, whereas functional phenotyping based on frailty and muscle strength provided substantially greater resolution in identifying clinically meaningful heterogeneity in perioperative vulnerability. Importantly, these observations were supported not only by descriptive cross-stratification analyses, but also by formal discrimination and regression analyses demonstrating superior classification performance of frailty-based approaches compared with age thresholds alone.

Chronological age, particularly when operationalized using a conventional ≥70-year threshold, showed poor discriminatory ability for prolonged hospitalization in our cohort. In contrast, frailty phenotype demonstrated improved classification performance, while the combined multivariable model integrating frailty, HGS, and clinical covariates achieved good discriminatory capacity. Moreover, chronological age was no longer independently associated with prolonged LOS after adjustment for frailty status and functional variables, whereas frailty remained strongly associated with postoperative burden. Collectively, these findings suggest that physiological reserve may represent a more informative dimension of perioperative vulnerability than chronological age alone.

The present findings are consistent with a substantial body of literature indicating that frailty captures dimensions of perioperative vulnerability not adequately represented by chronological age alone. Across surgical populations, age has repeatedly shown limited discriminatory capacity once functional and physiological factors are considered, whereas frailty status appears more closely aligned with postoperative burden.

Early work by Makary et al. [8] first demonstrated that a quantitative frailty index independently predicted postoperative morbidity and prolonged hospitalization beyond chronological age. Subsequent systematic reviews and meta-analyses reinforced these observations, reporting that age, when considered in isolation, often shows weak or inconsistent associations with postoperative outcomes, whereas frailty retains prognostic relevance [3,6,7]. More recent syntheses focusing on colorectal and general surgery populations have similarly suggested that chronological age performs poorly as a standalone risk discriminator, while frailty remains consistently associated with complications, extended LOS, and mortality [12,38].

Beyond confirming the limited utility of age-based stratification, our findings further illustrate the substantial overlap between chronological and physiological aging trajectories. In our cohort, a large proportion of older patients were classified as fit or pre-frail, whereas a clinically meaningful subset of younger individuals fulfilled frailty criteria and demonstrated elevated postoperative burden. Notably, frail patients younger than 70 years experienced markedly higher rates of prolonged hospitalization than fit older patients above the same age threshold. However, some subgroup estimates were derived from relatively small strata, particularly the younger frail subgroup, resulting in wider confidence intervals and limiting the precision of certain cross-stratum comparisons. Similar patterns have been reported in trauma and emergency surgery settings, where frailty in younger adults has been associated with risks comparable to, or exceeding, those observed in older non-frail individuals [13,14]. Conversely, studies in elective and oncologic surgery have suggested that functionally preserved older adults may tolerate major procedures with outcomes similar to those of younger patients when physiological reserve is maintained [2,20]. Together, these observations support the concept that chronological age alone incompletely captures perioperative resilience and functional heterogeneity across surgical populations.

Within this framework, our findings underscore the value of functional phenotyping not merely as a prognostic adjunct, but as a means of identifying clinically relevant heterogeneity within age strata. Frailty status and baseline HGS appeared to capture variability that remained obscured when patients were grouped according to chronological age alone. The wide dispersion and overlap of HGS values observed across age groups in our cohort align with previous population-based studies showing that muscle strength declines heterogeneously with aging and that substantial inter-individual variability persists even in advanced decades [21,22]. This heterogeneity likely reflects the cumulative interaction of physical activity, nutritional status, inflammatory burden, comorbidities, and sarcopenic trajectories, factors not adequately summarized by chronological age itself.

Consistent with previous literature, lower baseline HGS in our study tended to associate with greater postoperative burden, supporting the concept that grip strength may represent a pragmatic marker of physiological reserve rather than a purely geriatric descriptor [5,25,26,28,31]. Although HGS alone demonstrated only modest discriminatory performance, its inclusion within the multivariable functional model may provide complementary physiological information within integrated perioperative stratification frameworks beyond that captured by chronological age alone. Exploratory analyses using sex-specific HGS thresholds demonstrated a trend toward higher rates of prolonged hospitalization among patients with reduced muscle strength, although statistical significance was not reached. This finding may reflect both the multifactorial nature of postoperative recovery and the moderate sample size of the present cohort.

Importantly, HGS is closely linked to the broader concept of sarcopenia, which has emerged as a major determinant of perioperative vulnerability and adverse surgical outcomes [39]. Reduced muscle strength may reflect impaired muscle quality, diminished physiological reserve, and altered metabolic resilience associated with sarcopenic trajectories. Previous studies have consistently linked sarcopenia to prolonged hospitalization, increased postoperative complications, delayed functional recovery, and greater healthcare utilization across abdominal and oncologic surgery populations [40,41,42]. Although the present study did not include formal body composition assessment or standardized sarcopenia diagnostic criteria, the observed associations between lower HGS and postoperative burden further support the clinical relevance of muscle function assessment within contemporary perioperative evaluation frameworks.

An additional methodological consideration relates to the partial conceptual overlap between frailty phenotype and HGS. Within the phenotype-based frailty model, muscle weakness constitutes one of the defining domains and was operationalized using baseline HGS assessment. Consequently, some degree of shared variance between frailty status and HGS is expected. However, the two variables were not considered fully interchangeable constructs. Frailty phenotype reflects a broader multidimensional vulnerability framework incorporating multiple physiological and functional domains, whereas HGS provides a continuous quantitative measure of muscle performance and reserve. Multicollinearity diagnostics demonstrated low variance inflation factors for all covariates (all VIF values < 2), suggesting absence of problematic statistical collinearity within the adjusted model. Nevertheless, the independent contribution of HGS beyond frailty status should be interpreted cautiously and requires further validation in larger cohorts using alternative frailty operationalizations.

Our findings should not be interpreted as suggesting that chronological age lacks clinical relevance altogether. Age remains associated with biological decline, multimorbidity accumulation, and altered stress responses at the population level. However, the present data suggest that chronological age alone provides relatively limited discriminatory precision for individualized perioperative decision-making. The modest performance of age-based stratification observed in our analyses likely reflects the intrinsic heterogeneity of aging trajectories in contemporary surgical populations characterized by substantial variability in functional status and reserve.

It should also be acknowledged that the literature on frailty assessment is not entirely uniform. Some studies have reported modest or inconsistent performance of specific frailty instruments in predicting short-term postoperative complications. For example, Czajka et al. [43] found that several commonly used frailty scores demonstrated limited discriminatory ability for in-hospital complications after elective abdominal surgery, in some analyses performing no better than traditional risk indices. Such discrepancies may reflect differences in frailty operationalization, outcome definitions, cohort composition, or sample size, emphasizing that frailty is not a monolithic construct. In this context, the phenotype-based frailty model used in the present study, complemented by an objective continuous measure of muscle strength, may have enhanced sensitivity to functional vulnerability. Furthermore, by focusing on prolonged hospitalization as a cumulative marker of postoperative burden rather than a single binary complication endpoint, our analyses may have captured dimensions of recovery more closely related to physiological reserve and resilience.

Several strengths of this study merit consideration. The prospective multicenter design enhances generalizability across different institutional settings while reducing recall bias inherent to retrospective analyses. The use of standardized frailty criteria and objective dynamometric assessment of muscle strength provides reproducible and clinically feasible measures that can be integrated into routine perioperative workflows. In addition, the integration of descriptive stratification analyses with formal discrimination modeling allowed a more comprehensive evaluation of how chronological and functional variables classify perioperative risk.

At the same time, several limitations should be acknowledged. First, the cohort was restricted to patients undergoing elective abdominal surgery, and extrapolation to emergency procedures or other surgical specialties should be made cautiously. Second, prolonged LOS, while clinically meaningful, represents an indirect marker of postoperative burden and may be influenced by organizational or discharge-related factors beyond physiological recovery, including center-specific postoperative pathways, rehabilitation availability, operative approach, surgical complexity, and institutional discharge practices. Because the present multicenter cohort included heterogeneous abdominal procedures performed across different perioperative settings, residual confounding related to these factors cannot be entirely excluded. Accordingly, the present findings should primarily be interpreted as evidence of classification discordance and differential discriminatory performance rather than definitive proof of systematic clinical misclassification. Third, although the combined multivariable model demonstrated good discriminatory performance, the sample size and number of events remain moderate, and external validation in larger independent cohorts is warranted. Fourth, the phenotype-based frailty model employed in this study primarily captures physical dimensions of vulnerability; cognitive impairment, psychosocial support, and socioeconomic determinants were not systematically assessed and may provide additional explanatory value. In addition, some frailty domains were assessed using clinically pragmatic rather than instrument-based operational definitions, which may limit reproducibility across different perioperative settings. Finally, although the ≥70-year threshold reflects common clinical practice, alternative age cut-offs or continuous age-modeling strategies may yield different classification estimates. Accordingly, the present analyses should primarily be interpreted as evidence of differential discriminatory performance and clinically relevant classification discordance rather than definitive proof of causal risk misclassification.

## 5. Conclusions

From a clinical perspective, these findings support a broader shift toward function-centered perioperative assessment. Reliance predominantly on chronological age risks both overestimating vulnerability in functionally preserved older adults and under-recognizing risk in younger patients with impaired reserve. Incorporating frailty screening and objective functional measures such as HGS into routine preoperative evaluation may support more individualized risk discussions, facilitate allocation of perioperative resources, and help identify patients who may benefit from prehabilitation or intensified postoperative monitoring. In this regard, our findings align with contemporary geriatric surgery initiatives emphasizing the importance of “physiological age” and functional reserve over chronological age alone [11,20,34]. Future research should further investigate whether interventions targeting frailty and muscle function can modify perioperative trajectories and improve postoperative recovery. Integration of frailty and functional metrics into established surgical risk models also warrants further exploration to determine whether combined approaches consistently outperform age- or comorbidity-based strategies across different surgical populations. Longer-term follow-up studies assessing functional independence, quality of life, and survival may additionally help clarify the broader clinical implications of physiological versus chronological aging in perioperative medicine.

## Figures and Tables

**Table 1:** Baseline characteristics of the study population (n = 223).

Variable	Value
Age, years	64.7 ± 15.1
Female sex, n (%)	102 (45.7)
Male sex, n (%)	121 (54.3)
Oncologic indication, n (%)	118 (52.9)
Non-oncologic indication, n (%)	105 (47.1)
Fit phenotype, n (%)	83 (37.2)
Pre-frail phenotype, n (%)	91 (40.8)
Frail phenotype, n (%)	49 (22.0)
Baseline handgrip strength, kg	25.0 ± 11.0
Length of stay, days	7.8 ± 8.3
Prolonged LOS (>10 days), n (%)	48 (21.5)
Any postoperative complication, n (%)	80 (35.9)
Major postoperative complications (Clavien–Dindo ≥ III), n (%)	15 (6.7)

Data are presented as mean ± standard deviation or number (percentage), as appropriate.

**Table 2:** Cross-classification of patients by chronological age and frailty phenotype.

Age Group	Fit n (%)	Pre-Frail n (%)	Frail n (%)	Total
<70 years	64 (49.2)	56 (43.1)	10 (7.7)	130
≥70 years	19 (20.4)	35 (37.6)	39 (41.9)	93
Total	83 (37.2)	91 (40.8)	49 (22)	223

Data are presented as number (row percentage). Frailty status was defined using a phenotype-based model (fit = 0 criteria; pre-frail = 1–2 criteria; frail = 3–5 criteria). Age groups were defined using a conventional threshold of 70 years. This table illustrates discordance between chronological age and physiological vulnerability.

**Table 3:** Prolonged hospitalization across combined age–frailty strata.

Age–Frailty Stratum	Patients with Prolonged LOS/Total	Proportion (%)	95% CI
<70 years, fit	7/64	10.9	4.5–21.2
<70 years, pre-frail	14/56	25.0	14.4–38.4
<70 years, frail	4/10	40.0	12.2–73.8
≥70 years, fit	2/19	10.5	1.3–33.1
≥70 years, pre-frail	4/35	11.4	3.2–26.7
≥70 years, frail	17/39	43.6	27.8–60.4

Data are presented as absolute counts, proportions, and exact binomial 95% confidence intervals. Prolonged hospitalization was defined as LOS > 10 days.

**Table 4:** Discriminatory performance of chronological age, frailty phenotype, and handgrip strength for prolonged hospitalization (LOS > 10 days).

Variable/Model	AUC (95% CI)	Sensitivity (%)	Specificity (%)	PPV (%)	NPV (%)	Accuracy (%)
Chronological age (continuous)	0.579 (0.49–0.67)	—	—	—	—	—
Age ≥ 70 years	0.541 (0.45–0.63)	47.9	59.4	24.7	80.8	57.0
Frailty phenotype	0.679 (0.59–0.77)	50.0	86.3	49.0	86.7	78.5
Baseline HGS	0.544 (0.45–0.64)	—	—	—	—	—
Combined multivariable model ^†^	0.810 (0.73–0.89)	—	—	—	—	—

^†^ Combined model including chronological age, frailty phenotype, baseline handgrip strength, sex, and oncologic indication. For classification metrics (sensitivity, specificity, PPV, NPV, and accuracy), frailty-based stratification was operationalized using a dichotomous threshold corresponding to frail versus non-frail status, where frailty was defined as ≥3 phenotype criteria according to the phenotype-based frailty model.

**Table 5:** Multivariable logistic regression model for prolonged hospitalization (LOS > 10 days).

Variable	OR	95% CI	*p* Value
Chronological age (per year increase)	0.98	0.95–1.01	0.232
Frailty phenotype *	3.01	1.65–5.46	<0.001
Baseline HGS (per kg increase)	0.96	0.92–1.00	0.063
Female sex ^†^	0.22	0.08–0.60	0.003
Oncologic indication	9.01	3.54–22.96	<0.001

* Frailty phenotype was modeled as an ordinal categorical variable reflecting increasing physiological vulnerability (fit = 0, pre-frail = 1–2 criteria, frail = 3–5 criteria). ^†^ Sex was modeled as a binary covariate (male = reference category; female sex associated with lower odds of prolonged hospitalization). Baseline HGS and chronological age were modeled as continuous variables. Sex and oncologic indication were entered as binary covariates.

## Data Availability

The datasets generated and/or analyzed during the current study are not publicly available due to the presence of sensitive clinical information that could compromise participant privacy, but are available from the corresponding author upon reasonable request.

## Supplementary Materials

The following supporting information can be downloaded at: https://www.mdpi.com/article/10.3390/jcm15114187/s1, Table S1. Postoperative complications according to frailty phenotype.

## Abbreviations

The following abbreviations are used in this manuscript:

HGS	Handgrip strength
IQR	Interquartile range
LOS	Length of stay
STROBE	Strengthening the Reporting of Observational Studies in Epidemiology
