# Supplementary material for: Does Chronological Age Adequately Stratify Perioperative Risk? A Prospective Multicenter Cohort Study Using Frailty and Handgrip Strength
Source: J Clin Med. 2026 May 28;15(11):4187. doi: 10.3390/jcm15114187 (PMC13258233; doi:10.3390/jcm15114187)
Supplement: Supplementary file 1 [file jcm-15-04187-s001.zip › jcm-4324522-supplementary.pdf]

Supplementary Table S1. Postoperative complications according to frailty phenotype.

Frailty phenotype	Any postoperative complications n (%)	Major complications (Clavien–Dindo ≥III) n (%)
Fit (n = 83)	18 (21.7)	2 (2.4)
Pre-frail (n = 91)	33 (36.3)	5 (5.5)
Frail (n = 49)	29 (59.2)	8 (16.3)
Total (n = 223)	80 (35.9)	15 (6.7)

Data are presented as number (percentage). Postoperative complications were graded according to the Clavien–Dindo classification system.
